# 

**Published:** 1852-08

**Authors:** 


### NEW MEDICAL BOOKS.

We have received a number of new medical works which we shall be able to notice at length at an early day. The publishers, Blanchard & Lea, have issued a new edition of *Dr. Bartlett's Treatise on the Fevers of the United States.* We are often asked by students of medicine for the best work on this subject, and have no hesitation in referring them to this. They will be glad to learn that a new edition has been prepared by its accomplished author, and appears in the best style of its enterprising publishe s.

A new edition of *Wood's Practice* has also been issued, of which a review was prepared for the present number but was excluded by the press of matter on hand. It will appear in our next number.

Messrs. Blanchard & Lea have laid the profession under further obligations by new editions of *Meig's Treatise on Obstetrics,* and *Miller's Principles of Surgery,* both works of great ability, which have acquired a wide and enduring popularity.

The same publishers have issued the *Surgical Lectures of Bransby B. Cooper* in a style corresponding to the other publication of their house. It forms a handsome octavo volume of 759 pages, with an ample index, and may be confidently recommended to students and practitioners as a storehouse of reliable information on all Surgical topics.

*Analysis of Physiology* is the title of a small volume from the pen of *Dr. John J. Reese,* for which we are indebted to Messrs. Lindsay & Blakiston. It is designed to facilitate the study of this most pleasing branch of medical science, and is sure to find many purchasers among the numerous medical students who, in a few months, will be crowding the various schools of the country.

Dr. Horace Green, of New York, has made a valuable contribution to our professional literature in his little volume on the *Surgical Treatment of Polypi of the Larynx and Oedema of the Glottis,* a copy of which we have received through the publisher, G. P. Putnam, and which we shall take the earliest occasion to notice in detail.

### RECEIPTS OF MEDICAL JOURNAL FOR JULY, 1852.

| | |
|---|---|
| Dr. L. Lewis, Osceola, Mo., | $15 00 |
| —— Brookhart, Bardstown. Ky., | 5 00 |
| —— Dulaney, Blountsville, Tenn., | 5 00 |
| E. L. Lewis, Philadelphia. Miss., | 5 00 |
| B. L. Leavell. Oak Grove, Ky., | 5 00 |
| J. Windle, Oskaloosa, Iowa, | 5 00 |
| E. P Talbott. Waveland, Ind. | 2 00 |
| A. Ferring, Canadian, Miss., | 5 00 |
| S. B. Hobbs, Independence, Mo., | 10 00 |

The WESTERN JOURNAL OF MEDICINE and SURGERY is published monthly by the undersigned, at the corner of Main and Fifth streets, Louisville, at $5 per annum, payable in advance Each number contains 96 pages, making two volumes in the year of more than 550 pages each.

Contributions to its pages by the Physicians of the Valley of the Mississippi addressed to the Editors, are respectfully solicited.

Letters on business, to be addressed, postage paid, to the publishers.

PRENTICE & HENDERSON.

